# Specialist Dietary Intervention in Patients With Fibrotic Interstitial Lung Disease Experiencing Unintentional Weight Loss: A Pilot Randomized Controlled Trial

**DOI:** 10.1016/j.chest.2025.09.021

**Published:** 2025-09-25

**Authors:** Rasleen Kahai, Gioele Castelli, Fiammetta Danzo, Luis Ferreira, Arthihai Srirangan, Matteo Morviducci, Flavio Marco Mirabelli, Punchalee Kaenmuang, Cara Roberts, Simon Bax, Richard J. Hewitt, Maria Kokosi, Felix Chua, Vasileios Kouranos, Philip L. Molyneaux, Peter M. George, Richard Gisli Jenkins, Winston Banya, Carmel J.W. Stock, Steve Jones, Gemma Korff, Alastair Duncan, Athol U. Wells, Piersante Sestini, Elisabetta A. Renzoni

**Affiliations:** a Interstitial Lung Disease Unit, Royal Brompton Hospital, Guy’s & St Thomas’ NHS Foundation Trust, London, England; b Department of Nutrition and Dietetics, Royal Brompton Hospital, Guy’s & St Thomas’ NHS Foundation Trust, London, England; c Department of Cardiac, Thoracic, Vascular Sciences and Public Health, University of Padova, Padova, Italy; d Division of Respiratory Diseases, Luigi Sacco University Hospital, Milan, Portugal; e Unidade Local de Saude de Gaia e Espinho, Porto, Portugal; f Department of Public Health and Infectious Diseases, University of Rome La Sapienza, Roma, Italy; g Margaret Turner Warwick Centre for Fibrosing Lung Disease, National Heart and Lung Institute, Imperial College London, London, England; h Respiratory and Respiratory Critical Care Unit, Division of Internal Medicine, Faculty of Medicine, Prince of Songkla University, Songkhla, Thailand; j King's Centre of Lung Health, School of Immunology and Microbial Sciences, Faculty of Life Sciences and Medicine, King's College London, London, England; k European Pulmonary Fibrosis Federation, Brussels, Belgium; l Nutritional Sciences, King's College London, London, UK; m Respiratory Diseases, University of Siena, Siena, Italy

**Keywords:** antifibrotic drugs, BMI, dietary intervention, fibrotic interstitial lung disease, weight loss

## Abstract

**Background:**

Weight loss in patients with fibrotic interstitial lung disease (F-ILD) is associated with poor prognosis, yet the impact of dietary input is unknown.

**Research Question:**

What is the feasibility of a randomized controlled trial of specialist dietary intervention in F-ILD?

**Study Design and Methods:**

Patients with F-ILD experiencing low weight or weight loss were randomized 1:1 to either a 12-week specialist dietary intervention or a dietary information sheet by computer-generated sequence using random block design with stratification by antifibrotic drugs. The primary outcome was feasibility of recruitment, randomization, and retention. A key predefined exploratory outcome was weight change from baseline.

**Results:**

Of 128 screened patients, 40 patients (31%) were randomized, 19 to diet and 21 to control arm. The target number of patients was reached within 7 months, suggesting feasibility of a larger trial. All randomized patients completed the study. In the diet arm, 8 of 19 (42%) vs 1 of 21 (4.8%) participants in the control arm gained ≥ 1 kg at 12 weeks (OR, 14.2; 95% CI, 1.4-141.8; *P* = .02). Analysis of the exploratory outcome of weight change revealed that after a 4-week lag, the estimated rate of weight change between 4 and 12 weeks was –0.25 kg/month (95% CI, –0.61 to 0.12) in the control and +0.40 kg/month (95% CI, 0.02 to 0.78) in the diet group, with a difference of +0.65 kg/month (95% CI, 0.15 to 1.14) in favor of the diet group (*P* = .01).

**Interpretation:**

Based on the encouraging results of this pilot trial of dietary intervention in F-ILD, a definitive multicenter randomized controlled trial is warranted.

**Clinical Trial Registration:**

ClinicalTrials.gov; No.: NCT06016959; URL: www.clinicaltrials.gov)

**Take-Home Points**

**Research Question:** Is a randomized controlled dietary intervention trial in patients with fibrotic lung disease experiencing weight loss feasible?

**Results:** To our knowledge, this study is the first to show the feasibility of a dietary intervention trial in patients with fibrotic interstitial lung disease experiencing weight loss, and exploratory analyses suggest a beneficial impact on weight.

**Interpretation:** Based on the encouraging results of this pilot dietary intervention trial, a definitive multicenter randomized controlled trial is warranted.

Fibrotic interstitial lung diseases (F-ILDs) are characterized by major reduction in quality of life and shortened survival. Low BMI or unintentional weight loss has been associated with worse outcomes in patients with F-ILD.^1^, ^2^, ^3^

The pathophysiology of unintentional weight loss in F-ILD is not fully understood but is likely to be multifactorial, with contributors including increased work of breathing, inadequate food intake, increased catabolism, systemic inflammation, and physical inactivity.^4^ Gastrointestinal side effects from antifibrotic drugs also play a role, representing the most common cause of treatment discontinuation.^5^^,^^6^ In pirfenidone trials, patients who lost ≥ 5% of their body weight had poorer outcomes compared with those maintaining a stable weight.^7^ Weight loss during the first 6 months of nintedanib administration is an independent predictor of all-cause mortality.^8^

In other patient groups such as those with cancer and COPD, individualized dietary counseling by a dietitian has been shown to improve quality of life and energy intake, and to reduce weight loss when compared with standard care.^9^, ^10^, ^11^, ^12^, ^13^ However, despite the clear impact of low BMI/weight loss on outcomes, to our knowledge, no studies have evaluated the impact of dietary intervention in patients with ILD with low weight or unintentional weight loss. We therefore set out to perform a pilot study to assess the feasibility of a dietary intervention trial in patients with F-ILD and to explore potential impact on weight changes and GI symptoms.

## Study Design and Methods

This was a single-center 12-week randomized controlled parallel pilot study comparing a dietary intervention to a “poor appetite” information sheet (e-Fig 1). Patients were enrolled by the study dietitian after referral by ILD Unit clinical staff. All consecutive patients deemed likely to be eligible by their health care professional (physician, ILD specialist nurse, or ILD pharmacist) and interested in participating were referred to the specialist dietitian. Eligible participants were adult F-ILD patients with low weight or weight loss, as defined by at least 1 of the following criteria: (1) BMI ≤ 20 or if aged > 75 years, BMI ≤ 21; (2) unintentional weight loss ≥ 5% of body weight, regardless of baseline BMI over the past 12 months; (3) if BMI normal (18.5-25), unintentional weight loss > 2 kg over the past 12 months. Main exclusion criteria included (1) comorbidities currently requiring a specialized diet, including enteral feeding; (2) life expectancy < 6 weeks; (3) expected introduction of antifibrotic treatment or introduction/increase corticosteroid dose during trial period; (4) pregnancy; (5) comorbidities that could explain weight loss.

After a 2-week run-in period, participants were randomized by the clinical group’s statistician to dietary intervention or dietary information sheet, using a random block design with equal block distribution in a 1:1 allocation ratio within each stratum and block, using a computer-generated sequence, with stratification by use of antifibrotic drugs (pirfenidone or nintedanib) (e-Fig 1). Estimated daily patient calorie needs were calculated using the Henry equation.^14^ The same specialist dietician enrolled patients and carried out the dietary intervention. The dietary intervention included an initial consultation with the specialist respiratory dietitian, followed by nutritional counseling by the same dietitian during the scheduled follow-up contacts at 2, 4, and 8 weeks. Nutritional counseling included advice on food fortification and dietary habit, and where applicable a prescription request to the General Practitioner for oral nutritional supplements (ONSs). Further details on the visit setting and nutritional intervention are provided in the e-Appendix 1. Patients in the control arm were telephoned at the same time points to ask for their weight measurement, and they did not receive any nutritional counseling during the study but were provided with counseling by the same dietitian at the end of the 12-week period.

Because of the nature of the intervention, it was not possible to anonymize patients or dietitian, but input of outcome data in the clinical trial database was performed by independent researchers masked to the allocation arm.

In both intervention and control arms, weight was measured at the screening visit and at 2, 4, 8, and 12 weeks. Patients were asked to weigh themselves first thing in the morning with the same clothes on. The dietician conducted a training session on self-reported weight measurements during the first visit to ensure accurate measurements. Patients received VitaFit certified digital scales (accuracy, 0.05 kg) at their first visit or by home delivery. All weight measurements were taken by the patients weighing themselves each morning under consistent conditions and reporting their weight during scheduled calls.

At the screening visit and at the 12-week end of study timepoint, patients were asked to complete the following questionnaires: Gastrointestinal Symptom Rating Scale (GSRS),^15^ Patient-Generated Subjective Global Assessment short form (PG-SGA),^16^ the Hospital Anxiety and Depression Scale (HADS),^17^ the King’s Brief Interstitial Lung Disease Questionnaire (K-BILD),^18^ and the Clinical Frailty Scale (CFS).^19^ Patients were also asked to fill in a 3-day patient diary at baseline and in the final week of the trial. Average ingested daily calories were calculated using the software Nutritics. Further details on the questionnaires and diet diaries are provided in e-Appendix 1. At the 12-week visit, patients were asked whether overall they felt better, the same, or worse compared with at the screening visit.

The primary outcome was trial feasibility, including study recruitment, retention, and trial acceptability. The proportion of patients returning the 3-day diary and their quality were also evaluated as a secondary outcome linked to feasibility. A key predefined exploratory outcome was the change in weight at 2, 4, 8, and 12 weeks since baseline (in kg). Additional predefined exploratory outcomes included gastrointestinal- and respiratory-related quality of life, daily calorie intake, handgrip strength, and change in overall patient wellbeing. Although no formal implementation framework was adopted at the study design stage, outcomes related to acceptability and feasibility were pragmatically selected to reflect clinical applicability. Post-hoc mapping to the theoretical framework of acceptability (TFA)^20^ is considered in the Discussion section.

All patients provided written informed consent and ethical approval for the trial was obtained by the appropriate Research Ethics Committee (North East—Newcastle & North Tyneside 1 Research Ethics Committee): REC reference 23/NE/0093 (IRAS 327224). The trial was prospectively registered with ClinicalTrials.gov (NCT06016959). Patients (separate from those participating in the trial) were involved in study design and in designing the participant information sheet and consent form.

### Statistical Analysis

The planned feasibility sample was 40 patients, following sample size guidance for feasibility studies.^21^, ^22^, ^23^ Descriptive analyses were used for trial feasibility outcomes. Statistical methods used to analyze exploratory outcomes can be found in e-Appendix 1.

## Results

**Figure 1:**
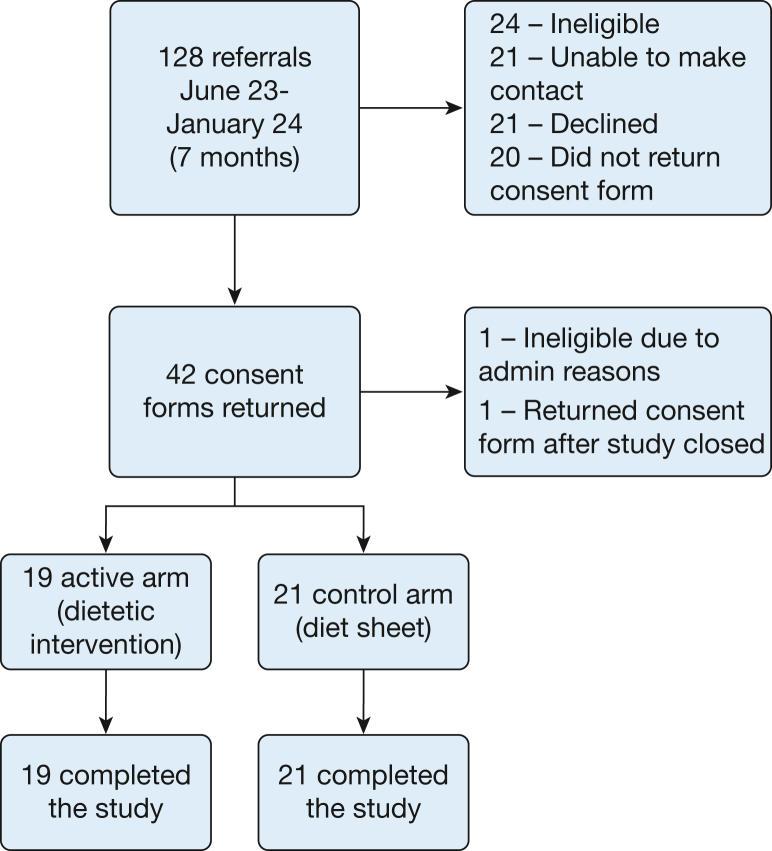
Patient flow diagram.

Between July 1, 2023 and December 31, 2023, 128 patients were screened. Of these, 40 were randomly assigned between July 14, 2023, and January 16, 2024, to the dietary intervention or the dietary information sheet (Fig 1). All 40 randomized patients completed the 12-week final study visit. All patients except for 2 in the control arm returned the baseline diet diary, and all subjects except for 3 in the control arm and 1 in the diet arm returned their 12-week diet diary. Information on completeness of diet diary entries is provided in e-Appendix 1. There were no missing data on weight at baseline or at 12 weeks. Six weight measurements were missing at intermediate time points, primarily because of patient unavailability caused by intercurrent hospitalizations in other facilities.

Baseline characteristics of patients according to treatment arm are summarized in Table 1. Mean weight loss in the year before trial entry was 7.7 kg (95% CI, 5.4-9.9) in the controls and 8.8 kg (95% CI, 5.9-11.6) in the dietary group, whereas mean percent weight loss was 10.3% (7.5%-13.2%) and 11.9% (8.5%-15.3%), respectively. Baseline characteristics were well balanced except for the K-BILD psychological and total scores, which were significantly lower (worse) in the control than in the dietary intervention arm. Three patients in each arm had a baseline BMI < 18.5. Thirteen patients in each arm (62% of control arm and 68% of intervention arm) were treated with antifibrotic drugs (5 pirfenidone, 21 nintedanib**)**. Baseline characteristics according to antifibrotic treatment are provided in e-Table 1. In the control group, 2 patients required antifibrotic dose reductions and 1 a 2-week break. In the dietetic group, no dose reductions were needed, but 4 patients took a 2-week break and 1 discontinued therapy. The proportion of patients with dose changes or interruptions did not differ between groups (14% in control group vs 26% in diet group; *P* = .44).

**Table 1:** Baseline Characteristics of Randomly Assigned Patients

Characteristic	Control Participant (n = 21)	Diet (n = 19)	*P* Value
Age, y	73.6 (70.4-76.7)	72.5 (69.3-75.7)	.63
Male sex	14 (67)	10 (53)	.37
Formerly smoked	11 (52)	10 (53)	.99
Diagnosis			
IPF	12 (57)	13 (68)	.52
HP	1 (5)	3 (16)	.33
PPFE (isolated)	2 (10)	2 (11)	.99
CTD-ILD	2 (10)	0 (0)	.49
Other^a^	4 (18)	1 (5)	.35
PPFE (with other ILD)	4 (19)	5 (26)	.73
BMI	21.7 (19.7- 24.4)	21 (19.7-24)	.58
BMI < 18.5	3 (14)	3 (16)	.90
FVC% predicted	70.5 (64.1-76.9)	71.2 (63.9-78.4)	.89
Dlco%	42.4 (33.0-51.9)	43.2 (36.1-50.4)	.89
CPI	52.8 (44.8-60.7)	50.4 (44.6-56.2)	.62
On antifibrotic drugs	13 (62)	13 (68)	.67
Weight loss 12 m, kg	7.7 (5.4–9.9)	8.8 (5.9–11.6)	.54
Weight loss 12 m, %	10.3 (7.4-13.3)	11.9 (8.4-15.4)	.48
Charlson Comorbidity Index	3.9 (3.2-4.5)	3.8 (3.2-4.4)	.97
PG-SGA	9.6 (6.4-12.7)	7.7 (5.2-10.3)	.38
HADS anxiety	7.6 (5.4-9.7)	5.7 (3.8-7.6)	.21
HADS depression	7.5 (5.5-9.4)	6.1 (4.1-8.0)	.31
GSRS abdominal pain	2.24 (1.70-2.77)	1.79 (1.46-2.11)	.17
GSRS reflux	1.95 (1.63-2.28)	1.77 (1.44-2.10)	.45
GSRS indigestion	2.27 (1.84-2.71)	2.03 (1.56-2.50)	.45
GSRS constipation	2.70 (1.98-3.42)	2.14 (1.59-2.69)	.24
GSRS diarrhea	3.05 (2.19-3.90)	2.30 (1.56-3.03)	.20
GSRS total	2.44 (2.01-2.88)	2.00 (1.66-2.35)	.13
Clinical frailty score	3.71 (3.14-4.29)	3.78 (3.30-4.28)	.85
Hand grip strength	19.8 (15.7-23.9)	21.5 (18.3-24.7)	.53
K-BILD psychological	45.4 (36.6-54.2)	59.1 (48.0-70.3)	.055
K-BILD dyspnea	35.3 (25.1-45.6)	44.1 (34.6-53.6)	.22
K-BILD chest symptoms	53.9 (42.4-65.3)	65.8 (55.8-75.8)	.13
K-BILD total	46.2 (38.4-53.9)	58.2 (49.2-67.1)	.046

Data are presented as No. (%) or mean (95% CI) assuming an approximate normal distribution, except for baseline BMI, which is presented as median (interquartile range [IQR]). CPI = composite physiologic index; CTD-ILD = connective tissue disease-related interstitial lung disease; Dlco = diffusing capacity of the lung for carbon monoxide; GSRS = Gastrointestinal Symptom Rating Scale; HADS = Hospital Anxiety and Depression Scale; HP = hypersensitivity pneumonitis; IPF = idiopathic pulmonary fibrosis; K-BILD = King’s Brief Interstitial Lung Disease Questionnaire; PG-SGA = Patient-Generated Subjective Global Assessment; PPFE = pleuro-parenchymal fibroelastosis.

a Other diagnosis include 2 lymphocytic interstitial pneumonia, 1 asbestosis, 1 combined pulmonary fibrosis and emphysema, 1 fibrosing organizing pneumonia.

### Weight Change

**Figure 2:**
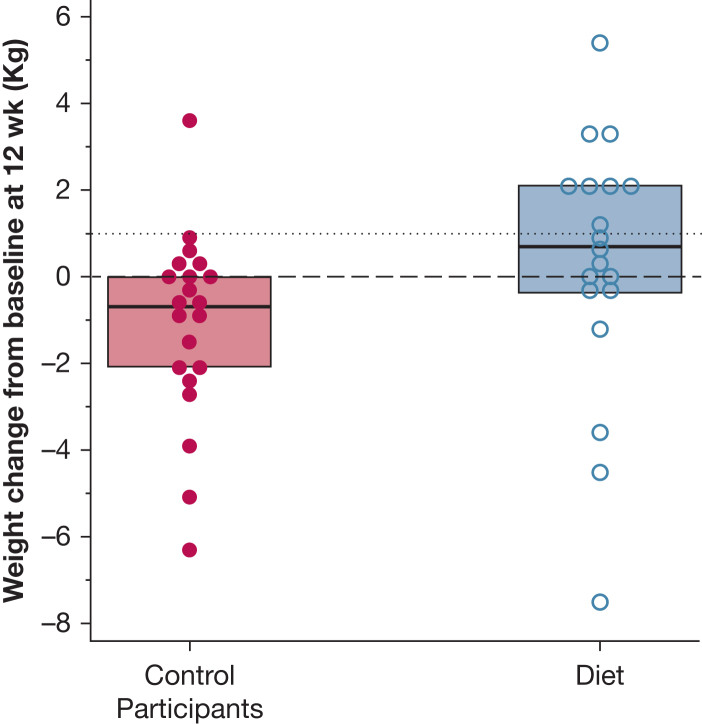
Strip plot of weight changes at 12 weeks. Symbols represent individual patients in the control group (left, solid circles) and in the dietary intervention group (right, hollow circles). Superimposed box plots represent the median and interquartile range of each group. A horizontal dotted line is drawn to mark an increase of 1 kg.

Weight change was a key predefined exploratory outcome in this pilot trial. In the diet arm, 8 of 19 (42.1%) vs 1 of 21 (4.8%) participants in the control arm gained at least 1 kg at 12 weeks (OR, 14.2; 95% CI, 1.4-141.8; *P* = .02, Fig 2). This difference remained statistically significant after adjustment for antifibrotic treatment (≥ 1 kg: OR, 17.3; 95% CI, 1.7-178.9; *P* = .017).

The median weight change from baseline at 12 weeks differed between the 2 groups, with –0.7 kg (interquartile range [IQR], –2.3 to 0.1) in the control group and +0.7 kg (IQR, –0.4 to 2.1) in the diet group (*P* = .019; Fig 2). This difference remained significant after adjusting for the use of antifibrotics using rank-based analysis of covariance (*P* = .015).

**Figure 3:**
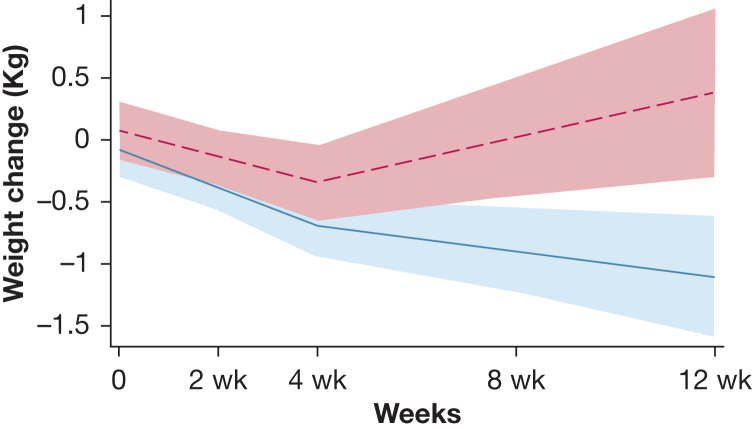
Mixed linear regression analysis of weight changes from baseline with random intercept and slope, using a linear spline with a single knot at 4 weeks. Solid line = controls. Dashed line = dietary intervention. Shaded areas represent the SE.

Analysis of weight changes over time (e-Fig 2) suggested a lag of approximately 4 weeks in dietary effect, after which the rate of weight change began to diverge between the 2 groups. Mixed linear regression analysis with random intercept and slope, using a linear spline with a single knot at 4 weeks (Fig 3), confirmed that the rate of weight change between time 0 and 4 weeks did not differ significantly between the groups: –0.65 kg/month (95% CI, –1.46 to 0.15) in controls, vs –0.50 kg/month (95% CI, –1.56 to 0.56) in the diet group (*P* = .822). In contrast, between 4 and 12 weeks, the estimated rate of weight change was –0.25 kg/month (95% CI, –0.61 to 0.12) in controls and +0.40 kg/month (95% CI, +0.02 to 0.78) in the diet group, with a difference of +0.65 kg/month (95% CI +0.15 to 1.14) in favor of the diet group (*P* = .01). This difference remained significant after adjusting for sex, age, baseline BMI, baseline composite physiologic index,^24^ smoking history, and the use of antifibrotics (*P* = .016).

### Patient Global Assessment

**Figure 4:**
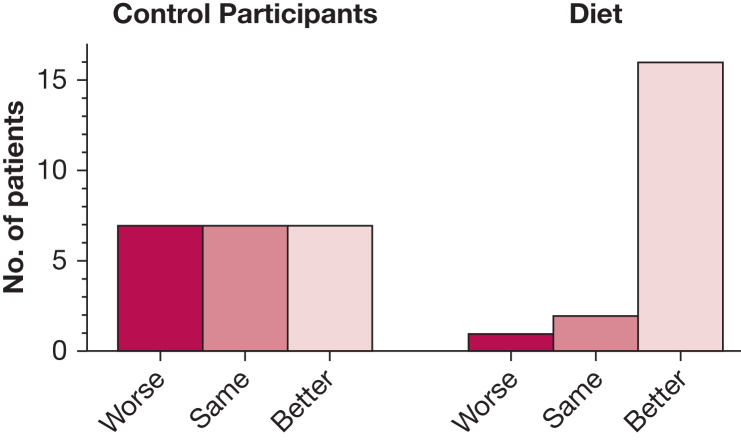
Numbers of patients reporting feeling overall better, the same, or worse after 12 weeks of dietary intervention or dietary information sheet.

At the end of the 12-week intervention, 16 of 19 patients in the diet group reported feeling better, 2 felt the same, and 1 felt worse, compared with 7 reporting feeling better, 7 the same, and 7 worse in the control group (*P* = .005), with an OR of 10.7 (95% CI, 2.3-49.3) for feeling better vs the same or worse (Fig 4).

### Symptom Questionnaires and Handgrip Strength

Questionnaire scores at 12 weeks, and the difference in score changes from baseline according to treatment arm, are summarized in Table 2. At 12 weeks, compared with control participants, there were no significant differences in the change from baseline for PG-SGA, GSRS, CFS, and HADS questionnaires. However, after adjustment for the respective baseline scores, GSRS total score and its subdomain constipation were significantly lower (less symptoms) in the diet arm compared with control participants (Table 2). Because of an imbalance between baseline K-BILD scores between the diet and control groups, the analysis was adjusted for respective baseline K-BILD scores. No significant difference was observed for any of the K-BILD total or subdomain scores once the analysis was adjusted for respective baseline scores. No differences were observed in the handgrip strength between the 2 groups.

**Table 2:** Assessment of Gastrointestinal Symptoms, Anxiety and Depression, Respiratory-Related Health Status, and Handgrip Strength After 12 Weeks of Dietary Intervention or Dietary Leaflet

Variable	Control Participants	Diet	Mean Between Treatment Difference in Change From Baseline	*P* Value	*P* Value^a^
GSRS (n = 36)					
Abdominal symptoms	2.4 (1.9 to 3)	1.7 (1.3 to 2.1)	–0.09 (–0.92 to 0.74)	.82	.13
Reflux	2 (1.5 to 2.5)	1.6 (1.2 to 2)	–0.15 (–0.77 to 0.47)	.64	.41
Indigestion	2.4 (1.9 to 2.9)	1.8 (1.4 to 2.3)	–0.19 (–0.84 to 0.45)	.56	.27
Constipation	3 (2.3 to 3.7)	1.5 (1.3 to 1.7)	–0.9 (–1.9 to 0.1)	.08	.001
Diarrhea	2.9 (2.1 to 3.6)	1.7 (1.2 to 2.3)	–0.13 (–1.18 to 0.92)	.81	.09
GSRS total	2.5 (2.1 to 2.9)	1.7 (1.4 to 2)	–0.29 (–0.8 to 0.21)	.26	.005
PG-SGA (n = 32)	7.4 (4.4 to 10.4)	4.4 (3.1 to 5.6)	–0.86 (–4.31 to 2.58)	.62	.19
CFS (n = 39)	3.8 (3.2 to 4.4)	3.7 (3.2 to 4.1)	0.26 (–0.04 to 0.56)	.09	.1
HADS (n = 34)					
Anxiety	7.9 (5.6 to 10.2)	4.8 (2.7 to 6.8)	–1.17 (–3.3 to 0.96)	.28	.15
Depression	8.2 (6.4 to 10)	5.4 (3.5 to 7.3)	–1.25 (–3.08 to 0.58)	.15	.05
K-BILD (n = 30)					
Psychological	58.2 (45.4 to 71)	56.9 (46.6 to 67.2)	–15.19 (–32.12 to 1.74)	.08	.30
Breathlessness	43.5 (29 to 57.9)	36 (24.4 to 47.6)	–12.7 (–26.13 to 0.72)	.06	.09
Chest symptoms	56.6 (42.4 to 70.9)	60 (47.9 to 72.1)	–7.81 (–21.3 to 5.68)	.26	.44
K-BILD total	55 (42.5 to 67.5)	53.3 (43.3 to 63.2)	–12.46 (–25.09 to 0.18)	.053	.12
Handgrip strength	21.2 (17.3 to 25.0)	22.4 (19.4 to 25.4)	–0.14 (–3.5 to 3.3)	.94	.86

Data are presented as mean (95% CI). CFS = Clinical Frailty Scale; GSRS = Gastrointestinal Symptom Rating Scale; HADS = Hospital Anxiety and Depression Scale; K-BILD = King’s Brief Interstitial Lung Disease Questionnaire; PG-SGA = Patient-Generated Subjective Global Assessment.

a Adjusted for respective baseline values.

### Patient Diet Diary-Derived Daily Calorie Intake

One patient did not return the baseline diet diary, and 4 patients did not return the 12-week diet diary. All patients in the diet group were prescribed ONSs, and all but 1 reported regularly taking the prescribed ONS at the follow-up telephone calls with the dietitian. At baseline, percent predicted recommended daily calorie intake was similar in the 2 groups: control participants, 90% (80%-100%); diet, 93% (84%-102%). At 12 weeks, it was 85% (74%-96%) in control participants and 104% (94%-113%) in the diet group, with a difference of 18% (1%-35%; *P* = .039), which remained significant after adjusting for baseline energy intake (*P* = .013).

### Adverse Events

The most common adverse events were GI symptoms. Serious adverse events were recorded in 9 patients in the diet and 8 in the control arm. All adverse events were considered unrelated to the trial (e-Table 2).

## Discussion

To our knowledge, this is the first study to examine the feasibility of a specialist dietary intervention in patients with F-ILD experiencing significant weight loss. This single-center pilot trial reached its recruitment target of 40 patients within only 7 months, suggesting the feasibility of such a trial, suitability of the recruitment strategies and eligibility criteria, and a high unmet need in this group of patients. Patient retention was excellent, with all patients completing the study. Although the primary outcome was feasibility, weight change was a key exploratory outcome in this pilot study. The specialist dietary intervention was associated with a reversal of the downward trend in weight, compared with ongoing weight loss in the control group. Confirmation of this preliminary finding in a large multicenter trial would support the integration of specialist ILD dietitians within routine care of patients with F-ILD. Despite considerable weight loss in the previous year, only 3 patients in each group had a BMI lower than 18.5, suggesting that weight loss and nutrition status should be actively assessed in all patients^4^ independently of a low BMI.

Significant differences in weight changes between the 2 groups were not seen during the first 4 weeks. This lag could be related to several factors, including delays in the communication with general practitioners regarding ONS prescription. Furthermore, there may be a physiologic lag in the time needed for a dietary change to impact weight.^25^ A similar delay of 4 weeks in weight benefits was seen in the control arm of a study investigating the effect of myostimulation, in which both arms received nutritional support.^26^^,^^27^

Antifibrotic agents nintedanib and pirfenidone can be associated with weight loss. Indeed, two-thirds of patients randomized to the trial were on antifibrotic agents, underscoring the need for close attention to weight loss in these patients. Patients who were not receiving antifibrotic treatment were more likely to be female and to have lower BMI, lower handgrip strength, higher levels of anxiety, and higher prevalence of pleuro-parenchymal fibroelastosis, a known risk factor for weight loss.^28^ Although this study was not sufficiently powered to evaluate the effect of the diet within treatment subgroups, adjusted analyses showed that antifibrotic treatment did not influence the difference in weight change between intervention and control groups, suggesting that the intervention was effective regardless of antifibrotic treatment. The average weight loss of 8 kg in the previous year is striking, as is the difference between patients receiving antifibrotic drugs (9.5 kg) and those not receiving antifibrotic drugs (5.8 kg). This highlights the severity of weight loss in a subgroup of patients with F-ILD, particularly those on antifibrotic drugs. Whether a preemptive dietary intervention in patients starting antifibrotic treatments can prevent the marked weight loss experienced by some patients will need to be investigated prospectively.

On univariable analysis, we did not observe significant differences in GI symptoms, although significant improvements in GSRS total score and in the constipation domain were observed after adjustment for the respective baseline scores. No significant differences were observed for the PG-SGA, the CFS, or the HADS, although there was a trend toward improvement in the HADS depression score in the diet group, once baseline scores were adjusted for. We also did not observe any changes in handgrip strength over this relatively short period.

The study has several limitations. The small sample of this pilot trial may have limited the power to detect an effect on patient-reported outcomes or handgrip strength. Furthermore, the duration was limited to 12 weeks. A longer duration may be necessary to identify changes to symptoms or handgrip strength in future randomized controlled trials. Furthermore, weight stabilization per se may not be sufficient to improve muscle strength, and a combined program of pulmonary rehabilitation with nutritional support may be needed to maximize improvements in muscle mass and strength, as suggested for patients with COPD.^29^ Although 84.2% of patients in the diet vs 33.3% in the control group reported feeling better at trial end, this outcome is subject to bias because the control group did not receive any intervention apart from the dietary information sheet. Because a dietary intervention could lead to increased energy or vitality and reduced fatigue, other outcome measures could be more sensitive to the dietary intervention and could be included in the larger trial, including a fatigue assessment scale,^30^ the Living With Pulmonary Fibrosis questionnaire,^31^ a visual analog scale to rate perceived energy, and a more generic health-related quality of life questionnaire such as the EQ-5D-5L.^32^ K-BILD scores were imbalanced between the 2 groups at baseline. However, once baseline scores were adjusted for, no significant difference in K-BILD scores was observed between the 2 groups. Inclusion was only based on weight and weight loss. In a large multicenter trial, it would be important to consider other factors of nutritional status, including malnutrition risk scores assessed specifically in ILD.^33^^,^^34^ Finally, there are potential limitations in the validity of self-reported weight. Previous studies have reported reasonable accuracy in self-reported weights, including among postpartum individuals^35^ and general practice patients, in whom intraclass correlations exceeded 0.9 despite individual discrepancies.^36^ Accuracy did not improve when patients knew their weight would be measured. In a larger trial, we would plan to have digital transmission of weights directly from the scale and to validate self-reported weights against centrally measured values in a validation subgroup.

Because of the nature of the intervention, it was not possible to anonymize trial participants or the dietitian conducting the study. Data entry for clinical trial outcomes was done by assessors masked to trial information, and the patients completed questionnaires independently. However, we cannot exclude bias in the study results, because the unmasked intervention may have inflated self-reported benefits.

The dietary intervention was conducted by a single highly experienced dietitian specializing in treating respiratory patients. A large multicenter trial is needed to assess generalizability across different respiratory services. Furthermore, a future trial could assess which aspects of the dietary intervention are most effective and could include an arm with dietary supplement and with limited or no dietician intervention, to be compared with an arm containing the full dietician intervention, including the dietary supplements.

Although the end-of-study outcomes weight and patient global assessment were available for all patients, because they were collected during the last trial visit via video or over the phone, questionnaires were completed at home on a paper copy, with several questionnaires not returned via the post. Most of the patients do not live in close proximity to the hospital, and patients had the choice not to return to the hospital in person for their final visit if not convenient, because most dietary care is not routinely face-to-face in clinical practice in the United Kingdom. This resulted in a lower degree of control on questionnaire return, with a minority not returning some or all the final questionnaires despite multiple reminders. For a larger trial, we would consider online collection with a tablet or other web-based platform. Compared with paper-based questionnaires, online administration of Patient Reported Outcome Measures (PROMs) leads to better data quality, faster completion, and reduced missing values.^37^ However, although studies have found that online administration of PROMs is feasible in older adults, several patients included in the study had to ask younger family members to help with access to online video consultations. Using solely an online application could have led to the exclusion of the more digitally naive older patients. A hybrid paper/online collection of PROMs according to patient choice is a possible solution, with paper forms collected over the telephone by an independent researcher masked to trial intervention to ensure timely completion. Finally, food diary completion was suboptimal, with diaries not returned in 5 cases, and poorly filled out by some patients. Alternative ways to measure dietary intake should be further explored with patients for future trials. These could include food intake photography to verify and enhance the accuracy of food diary completion. Corrections for multiple outcome comparisons were not performed in view of the exploratory nature of this pilot trial. We did not investigate body composition and how this changed during the course of the dietary intervention. In the larger multicenter trial, we would plan to perform a detailed assessment of the participants’ nutritional status, including body composition analysis by bioimpedance analysis, serological markers of nutritional status, as well as ultrasound assessment of muscle mass at baseline and on trial completion to understand muscle-specific impacts of a dietary intervention.

A further limitation of the study is the lack of a formal implementation outcomes framework to guide the selection and interpretation of feasibility-related outcomes. Recruitment, retention, and global patient assessments were used as proxies for acceptability, but these are not comprehensive. In hindsight, several of our measures align with domains from the Theoretical Framework of Acceptability (TFA).^20^ For example, “affective attitude” and “perceived effectiveness” were indirectly reflected in the patient global assessment, “burden” could be inferred from retention rates and questionnaire/diary completion, and “intervention coherence” was improved by involving patients in the design of the study as well as in the design of the participant information sheet and the consent form. Although retention was excellent, questionnaire and diary completion rates were suboptimal, as discussed previously, indicating the need for improved methods of capturing these outcomes in a way that is acceptable to patients. In future studies, we plan to incorporate a formal implementation framework to enable a systematic evaluation of acceptability for both intervention providers and recipients, and to assess adoption, fidelity, and sustainability.^20^ This will include qualitative inquiry as part of a mixed-methods approach in a future randomized controlled trial, using the TFA to structure topic guides for questionnaires, interviews, and focus groups.

## Interpretation

In conclusion, this study is the first to our knowledge to show feasibility data for conducting a randomized controlled trial of a specialist dietary intervention in patients with F-ILD experiencing significant weight loss. In this pilot trial, dietary intervention appeared to be associated with prevention of ongoing weight loss. A definitive randomized controlled trial of dietary intervention in patients with F-ILD is now required to further explore the impact on weight loss and assess any additional treatment effects on meaningful longer-term outcomes.

## Funding/Support

Funding for the trial was provided by the Royal Brompton & Harefield Hospitals Charity (Grant No.: R403).

## Financial/Nonfinancial Disclosures

The authors have reported to *CHEST* the following: R. K. reports support from a research fellowship from the Royal Brompton and Harefield Hospital Charity, lecture fees from Boehringer Ingelheim, and is a Trustee (Director) of the Alexandra Rose Charity. S. B. reports personal lecture fees from Boehringer Ingelheim. R. H. reports grants held by Kings’ College London from Asthma + Lung UK Early Career Starter Grant (ECSG24\36), King’s Health Partners Centre for Translational Medicine Translational Medicine Pilot Award, and Sir Robert Finch Fellowship’ from the Royal Brompton and Harefield Hospitals Charity, and personal speaker fees from Boehringer Ingelheim. M. K. reports honoraria from Roche and Boehringer Ingelheim. F. C. reports honoraria from Boehringer-Ingelheim for lectures, educational events, and meetings, payments for expert advisory meetings (unrelated to technology appraisals) from NICE (UK), support for travel costs form Boehringer-Ingelheim, and unpaid committee work from the British Thoracis Society and the European Respiratory Society. V. K. reports lecture fees from Boehringer Ingelheim. P. L. M. reports grants paid to his institution from AstraZeneca, GalaxoSmithKline, Asthma & lung UK, and Action for Pulmonary Fibrosis, advisory board fees from Hoffman-La Roche, Boehringer Ingelheim, AstraZeneca, Trevi, Qureight, and Endevour, lecture fees from Boehringer Ingelheim, and Hoffman-La Roche, board participation fees paid directly from United Therapeutics, and stock options in Qureight. P. M. G. reports research grants paid to his institution from Boehringer Ingelheim, lecture fees from Boehringer Ingelheim, Roche, Teva, Cipla, Brainomix, AstraZeneca, Daiichi-Sankyo, and Avalyn, support for conference attendance from Boehringer Ingelheim and Roche, advisory board fees from GalaxoSmithKline, and stock options for, and is Senior Medical Director of, Brainomix. R. G. J. reports grant payments to his institution from Astra Zeneca, Galecto, GalaxoSmithKline, Nordic Biosciences, RedX, and Pliant, consulting fees from AbbVie, AdAlta, Apollo Therapeutics, Arda Therapeutics, Astra Zeneca, Brainomix, Bristol Myers Squibb, Chiesi, Cohbar, Galecto, GlaxoSmithKline, Mediar Therapeutics, RedX, Syndax, and Pliant, lecture fees from Boehringer Ingelheim, Chiesi, Roche, and AstraZeneca, payment for expert testimony from Pinsent Masons LLP, participation on advisory boards for Boehringer Ingelheim, Galapagos, and Vicore, is President of Action for Pulmonary Fibrosis, and board member for NuMedii, and is the Chair of Editorial Board for BMJ Open Respiratory Research. S. J. reports consulting fees paid to the European Pulmonary Fibrosis Federation for Short consultancy assignments with a range of pharmaceutical organisations on the PF patient journey and patient involvement in clinical trials, payments via the European Pulmonary Fibrosis Federation for travel and accommodation for attendance at 3 pharma companies’ Patient Advisory Groups, of which he is a member, and payments for membership of 2 Data Safety Monitoring Board Committees, details of which are confidential. A. D. reports research awards paid to institution from National Institute for Health Research, King’s Health Partners Academic Health Sciences Centre, and South East London NHS Integrated Care Board, and personal honoraria fees from Gilead Pharmaceuticals. A. U. W. reports consulting fees from Boehringer Ingelheim, Roche, Veracyte, and Chiesi, lecture fees from Boehringer Ingelheim, and is the President of WASOG. E. A. R. reports advisory board and lecture fees from Boehringer Ingelheim and Roche, and lecture fees from Mundipharma, paid to her institution. None declared (G. C., F. D., L. F., A. S., M. M., F. M. M., P. K., C. R., W. B., C. J. W. S., G. K., and P. S.).

## Supplementary Data

e-Online Data
