# Supplementary material for: Specialist Dietary Intervention in Patients With Fibrotic Interstitial Lung Disease Experiencing Unintentional Weight Loss: A Pilot Randomized Controlled Trial
Source: Chest. 2025 Sep 25;169(3):687–97. doi: 10.1016/j.chest.2025.09.021 (PMC12975389; doi:10.1016/j.chest.2025.09.021)
Supplement: e-Online Data [file mmc1.doc]

**Specialist dietetic intervention in patients with fibrotic interstitial lung disease experiencing unintentional weight loss: a pilot randomised controlled trial**

**METHODS**

**Setting of clinical trial visits**

Dietitian reviews are often done remotely in our hospital, either via video or over the telephone. In this trial, the screening visit was carried out face to face in six patients, via video call in eight patients, and via telephone in seven patients in the control group, while in the diet group it was carried out in person in four, via video in twelve and via telephone in three patients. The first dietary intervention visit was carried out solely in the diet group and was in person for three patients, via telephone for seven and via video for nine patients. The 12-week visit was carried out face to face in four patients, via video call in four patients and via telephone in 13 patients in the control group, while it was carried out in person in two, via video in nine and via telephone in eight patients in the diet group. The scheduled follow-up contacts at 2,4, and 8 weeks were all performed via telephone except for one patient in the control arm at the week 4 visit, who had an in-person follow-up as they were in the hospital for another appointment.


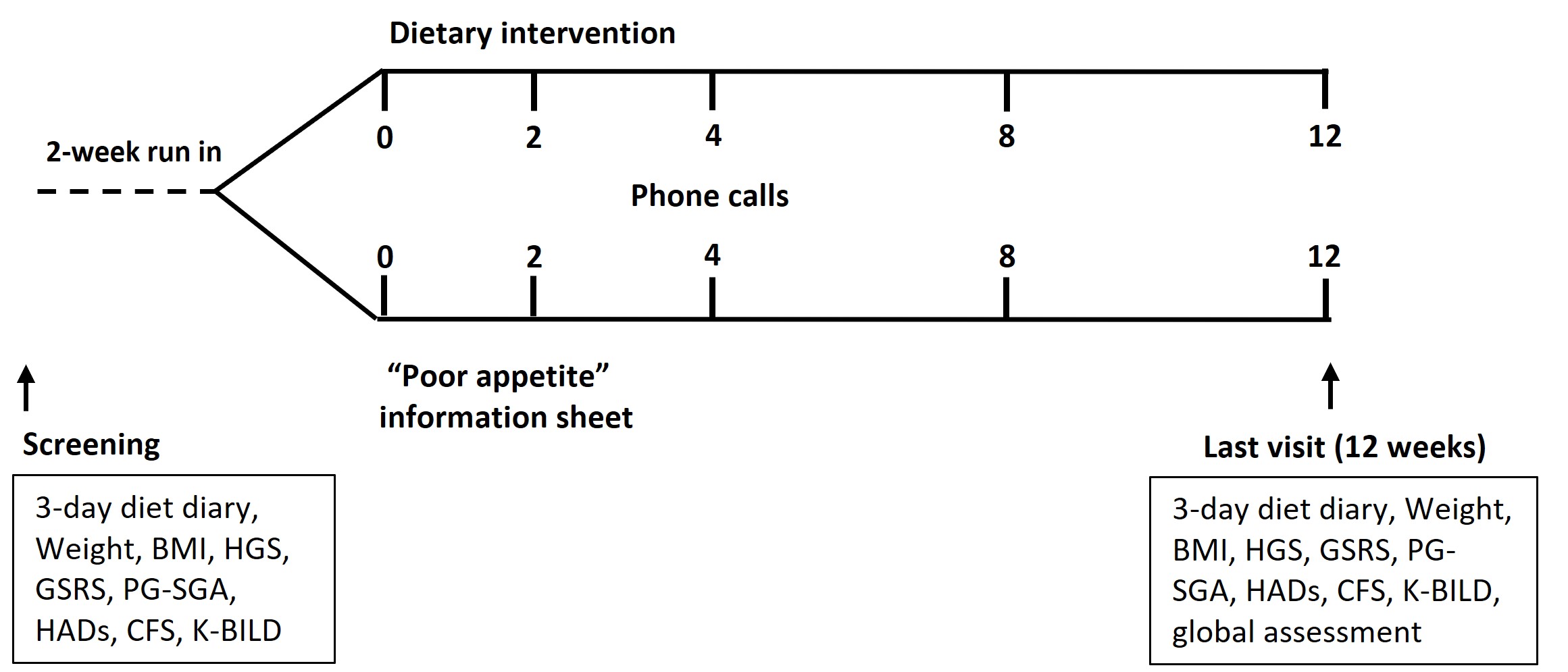


**Supplementary e-Figure 1. Study design schematic.**

**Nutritional intervention**The dietary intervention included individualized dietary advice looking at each patient’s individual goals, and primarily aimed at increasing overall energy and nutrient intake and improving symptom control, by providing advice on food fortification, improvement of dietary habit, and where applicable, a prescription request of oral nutritional supplements (ONS). ONS provide a variety of micro and macronutrients, providing an extra 600-700kcal/day and a minimum of 24g protein per day. Patients were provided with different flavor samples to choose from, and the patient’s GP was then asked to prescribe the ONS, with an average 2-week delay in the ONS prescription.  Symptom control advice included holistic nutritional advice to improve primarily gastro-intestinal (GI) symptoms, as well as liaising with multidisciplinary team members to support medication changes to improve symptoms. Advice included suggestions on how to counteract bloating, including use of peppermint capsules, counselling on loperamide use, reflux optimization, as well as dietary advice on nausea and constipation, individualized to the participant, depending on symptoms.

**Questionnaire description**

The GSRS is a widely validated gastro-intestinal symptom questionnaire with 7-day recall, consisting of 15 items combined into five symptom clusters relating to reflux, abdominal pain, indigestion, diarrhoea, and constipation, graded on a Likert-type scale from 1 (no symptoms) to 7 (very troublesome symptoms) [1]. The PG-SGA asks 4 questions about weight history in the past 6 months, 2 months, and 2 weeks; food intake in the past month; symptoms that kept participants from eating in the past 2 weeks; and changes in daily activities. Higher scores indicate worse nutritional status [2]. The HADS consists of 14 items, seven for depression and seven for anxiety, with scores on each subscale ranging from 0 to 21, with higher scores indicating worse symptoms [3]. The K-BILD comprises 15 questions, each with a seven-point response scale, grouped into three domains: breathlessness and activities, chest symptoms, and psychological symptoms, with lower scores indicating worse ILD-related health status [4]. The Clinical Frailty Scale (CFS) has been developed in elderly patients to summarise overall frailty or fitness. It is a 9-point scale which ranges from 1 (very fit) to 4 (vulnerable/living with very mild frailty) to 9 (terminally ill) [5]. Daily calorie intake was calculated from the three-day patient diaries (at baseline and 12 weeks) using Nutritics software. In the diaries, patients were asked to list in detail the foods, and their quantities, eaten throughout the day, including all three main meals and any snacks. As seven patients did not consider ONS as food, and therefore did not include ONS in the diaries, the calculation of daily calorie intake was integrated with information on ONS intake from the trial visit transcripts. Overall patient-reported change in well-being was evaluated by asking the question: “Since the start of the study, overall do you feel better, the same or worse?”

**Statistical analysis**

**The Wilcoxon rank-sum test was used to compare the median change in weight at 12 weeks compared to baseline.** Adjustment for antifibrotic treatment was performed using rank-based ANCOVA, i.e. linear regression on the ranked outcome, including treatment arm and antifibrotic use as covariates [6]. **Six weight measurements were missing at intermediate time points, primarily due to patient unavailability caused by intercurrent hospitalisations in other facilities. These missing values were imputed using multiple imputation based on regression, with 20 replicates. Weight changes from baseline over time were analysed using mixed linear models, with the patient included as a random effect. The difference in the rate of weight change between the two experimental groups was assessed by evaluating the interaction term between treatment and time.** Comparisons between diet and control arms in patients gaining at least 1kg in weight at the end of the trial, as well as in patients reporting feeling better, compared to those reporting stability or worsening at the end of the trial, were performed by logistic regression.

The difference between baseline and end trial questionnaire scores was evaluated by generalized linear models, using Gaussian or binomial families, as appropriate. Differences in frequencies were compared by using Fisher’s exact or chi square tests, as appropriate. Results are presented as the mean or OR with 95% CI, or as the median with IQR, as appropriate. Level of statistical significance was set at p<0.05. Any p-values presented for these exploratory endpoints were considered nominal in nature and no adjustment for multiplicity was made. **Statistical analysis was performed using Stata 18 for Windows, StataCorp, College Station, TX.**

**RESULTS**

**Baseline comparison between patients with and without anti-fibrotic treatment**

Compared to patients on treatment with anti-fibrotic drugs, individuals not on anti-fibrotic treatment were more likely to be female, had lower BMI but had lost less weight over the previous 12 months, had lower handgrip strength, and higher HADS anxiety scores. PPFE was present in 8 out of 14 patients (57.1%) who were not on anti-fibrotic treatment, either isolated PPFE in 4 patients or associated with a separate ILD process, with 7/8 cases being female, compared to 5 patients with PPFE-associated with a separate ILD process (19.2%) in the group on anti-fibrotics, with 3/5 being male.

**e-Table 1 – Baseline characteristics of the trial population, according to antifibrotic treatment**

|  | **Total cohort**  **(n=40)** | **Untreated**  **(n=14)** | **Treated**  **(n=26)** | **p value** |
| --- | --- | --- | --- | --- |
| **Female n - %** | 16 (40) | 11 (78.6) | 5 (19.2) | **<0.001** |
| **Age – years** | 73.1 (70.8-75.3) | 71.4 (68.5-74.3) | 73.9 (70.9-77.0) | 0.30 |
| **Diagnosis** |  |  |  |  |
| - **IPF** | 25 (62) | 5 (36) | 20 (77) | **0.02** |
| - **HP** | 4 (10) | 1 (7) | 3 (12) | 0.99 |
| - **CTD-ILD** | 2 (5) | 1 (7) | 1 (4) | 0.99 |
| - **PPFE (isolated)** | 4 (10) | 4 (29) | 0 (0) | **0.02** |
| - **Other*** | 5 (13) | 3 (21) | 2 (8) | 0.32 |
| - **PPFE with separate ILD** | 9 (22.5) | 4 (29) | 5 (29) | 0.69 |
| **Weight – kg** | 63.5 (59.4-67.6) | 55.3 (50.7-59.8) | 67.9 (62.8-73.0) | **0.001** |
| **Weight loss (kg) over previous year - %** | 8.2 (6.4-10.0) | 5.8 (3.7-7.9) | 9.5 (7.1-11.8) | **0.046** |
| **BMI** | 22.0 (21.0-23.1) | 20.0 (18.8-21.2) | 23.1 (21.8-24.5) | **0.004** |
| **Smoking status** |  | | | |
| ***Former n - %*** | 21 (53) | 5 (36) | 16 (62) | 0.19 |
| ***Never n - %*** | 19 (47) | 9 (64) | 10 (38) |
| **Pack years – n** | 12 (9.5) | 12.3 (7.6) | 12 (12) | 0.69 |
| **Charlson Comorbidities Index** | 3.8 (3.4-4.3) | 3.4 (2.8-4.0) | 4.1 (3.5-4.7) | 0.17 |
| **Clinical frailty score** | 3.8 (3.4-4.1) | 3.7 (3.0-4.4) | 3.8 (3.3-4.2) | 0.89 |
| **Mean Handgrip** | 20.6 (18.0-23.2) | 16.0 (11.7-20.3) | 23.1 (20.2-26.0) | **0.009** |
| **Baseline questionnaires** | | | | |
| **PG-SGA** | 8.7 (6.6-10.7) | 7.2 (4.3-10.2) | 9.4 (6.7-12.1) | 0.40 |
| **K-BILD Psychological** | 51.7 (44.5-59.0) | 45.7 (33.4-58.0) | 55.1 (46.3-64.0) | 0.15 |
| **K-BILD Dyspnea** | 39.4 (32.3-46.5) | 40.9 (29.8-52.0) | 38.6 (29.2-47.9) | 0.91 |
| **K-BILD Chest symptoms** | 59.4 (51.5-67.2) | 49.1 (34.8-63.4) | 65.1 (56.5-73.8) | 0.08 |
| **K-BILD total** | 51.7 (45.6-57.8) | 46.7 (36.2-57.2) | 54.5 (47.1-61.9) | 0.14 |
| **HADS Anxiety** | 6.6 (5.2-8.1) | 8.7 (6.2-11.3) | 5.5 (3.8-7.1) | **0.04** |
| **HADS Depression** | 6.8 (5.4-8.1) | 7.5 (5.1-9.9) | 6.4 (4.7-8.1) | 0.53 |
| **GSRS abdominal pain** | 2.0 (1.7-2.3) | 2.3 (1.6-3.0) | 1.9 (1.6-2.2) | 0.48 |
| **GSRS reflux** | 1.9 (1.6-2.1) | 2.0 (1.6-2.4) | 1.8 (1.5-2.1) | 0.25 |
| **GSRS indigestion** | 2.2 (1.8-2.5) | 2.3 (1.7-2.8) | 2.1 (1.7-2.5) | 0.50 |
| **GSRS constipation** | 2.4 (2.0-2.9) | 2.0 (1.2-2.8) | 2.7 (2.1-2.3) | 0.18 |
| **GSRS diarrhoea** | 2.7 (2.1-3.3) | 2.3 (1.4-3.2) | 2.9 (2.2-3.6) | 0.41 |
| **GSRS Total** | 2.2 (1.9-2.5) | 2.2 (1.6-2.7) | 2.3 (1.9-2.6) | 0.73 |
| **Baseline Pulmonary Function Tests** | | | | |
| **FVC%** | 70.8 (66.1-75.6) | 73.3 (66.4-80.1) | 69.5 (63.2-75.8) | 0.60 |
| **FEV1 %** | 74.9 (70.3-79.5) | 75.0 (68.0-81.9) | 74.9 (68.7-81.0) | 0.85 |
| **DLCO%** | 42.8 (37.3-48.3) | 48.3 (39.0-57.7) | 39.8 (33.2-46.5) | 0.23 |
| **Baseline Diet Intake** | | | | |
| **Kilocalories intake** | 1708 (1593-1823) | 1602 (1471-1732) | 1763 (1604-1921) | 0.25 |

Data are mean (95% C.I.) or n (%). *Other diagnosis include: 2 lymphocytic interstitial pneumonia, 1 asbestosis, 1 combined pulmonary fibrosis and emphysema, 1 fibrosing organizing pneumonia. BMI – body mass index; CTD-ILD – Connective tissue disease related interstitial lung disease; DLCO- Lung Diffusion of Carbon Monoxide; FEV1 - Forced Expiratory Volume 1^st^ second; FVC – Forced Vital Capacity; GSRS - Gastrointestinal Symptom Rating Scale; HADS - Hospital Anxiety and Depression Scale; IPF=idiopathic pulmonary fibrosis; HP=hypersensitivity pneumonitis; K-BILD - King’s Brief Interstitial Lung Disease; PG-SGA - Patient-Generated Subjective Global Assessment; PPFE= pleuro-parenchymal fibroelastosis.

**Evaluation of diet diary completion**

The three-day diet diaries were independently evaluated for level of completeness by two independent researchers blind to allocation arm. Completeness of patient diet diaries was categorised in a semiquantitative manner as very good, moderate, or poor, depending on the detail provided (number of meals completed for all three days, the degree to which amount for each food was specified). In the diet group, seven patients did not report ONS in the patient diaries while regularly taking them as reported at the scheduled clinical trial visits.

At baseline, 19/21 of patients in the control arm returned the diet diaries. Of these, level of completeness was very good for 14 and moderate for 5. In the diet arm, all 19 patients returned their baseline questionnaire, with completeness categorised as very good for 14, moderate for 3, and poor for 1. At 12 weeks, three patients in the control arm, and one in the diet arm did not return the diet diaries. Completeness was very good in 12, moderate in 3, and poor in 4 patients in the control arm, and very good in 10, moderate in 6 and poor in 2 in the diet arm. Overall there was a trend towards a higher number of patients completing the diet diaries poorly at 12 weeks, although this did not reach statistical significance (p=0.1).


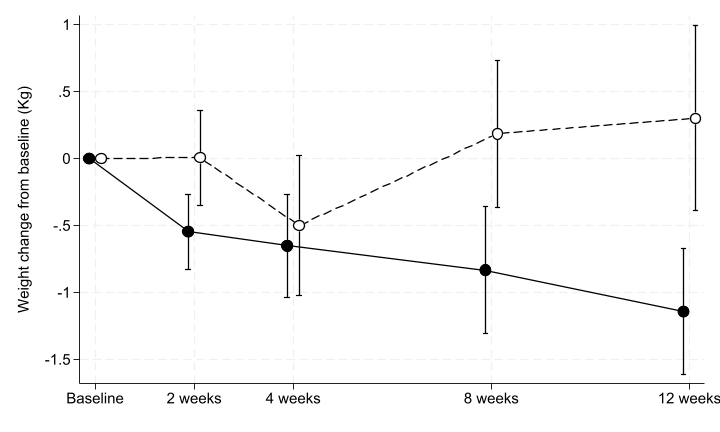


**Supplementary e-Figure 2. Weight changes from baseline over time in the two groups.**

The solid line with black circles represents the control group, while the dashed line with white circles represents the dietary intervention group. Error bars indicate the SE.

**e-Table 2. Adverse Events**

| **Event** | **Diet Group (n=19)** | **Control Group (n=21)** |
| --- | --- | --- |
| **Mild to Moderate AE:** | | |
| **Sore Nose** | 0 (0%) | 2 (10%) |
| **Sore Throat** | 0 (0%) | 1 (5%) |
| **Light Headache** | 1 (5%) | 0 (0%) |
| **Itchiness** | 0 (0%) | 0 (0%) |
| **Skin rash** | 1 (5%) | 1 (5%) |
| **UTI infection** | 1 (5%) | 1 (5%) |
| **Gastrointestinal symptoms** | 14 (74%) | 16 (76%) |
| **Reflux** | 4 (21%) | 6 (29%) |
| **Diarrhoea** | 9 (47%) | 10 (48%) |
| **Vomit** | 3 (16%) | 2 (10%) |
| **Nausea** | 4 (21%) | 7 (33%) |
| **Other** | 3 (16%) | 5 (24%) |
| **Upper Respiratory tract infection** | 3 (16%) | 2 (10%) |
| **Severe AE:** | | |
| **Pneumonia** | 2 (11%) | 2 (10%) |
| **Unstable respiratory symptoms** | 4 (21%) | 4 (19%) |
| **Chest pain*** | 1 (5%) | 2 (10%) |
| **Acute exacerbation ILD** | 2 (11%) | 0 (0%) |

*cardiac origin subsequently excluded by appropriate investigations

1. Dimenas E, Glise H, Hallerback B, et al. Well-being and gastrointestinal symptoms among patients referred to endoscopy owing to suspected duodenal ulcer. Scand J Gastroenterol. 1995;30(11):1046-52.

2. Ottery FD. Definition of standardized nutritional assessment and interventional pathways in oncology. Nutrition. 1996;12(1 Suppl):S15-9.

3. Zigmond AS, Snaith RP. The hospital anxiety and depression scale. Acta Psychiatr Scand. 1983;67(6):361-70.

4. Patel AS, Siegert RJ, Brignall K, et al. The development and validation of the King's Brief Interstitial Lung Disease (K-BILD) health status questionnaire. Thorax. 2012;67(9):804-10.

5. Rockwood K, Song X, MacKnight C, et al. A global clinical measure of fitness and frailty in elderly people. CMAJ. 2005;173(5):489-95.

6. Conover WJ, Iman RL. Analysis of covariance using the rank transformation. Biometrics. 1982;38(3):715-24.
